# Stability and square integrability of derivatives of solutions of nonlinear fourth order differential equations with delay

**DOI:** 10.1186/s13660-017-1353-8

**Published:** 2017-06-09

**Authors:** Erdal Korkmaz

**Affiliations:** Mathematics, Faculty of Arts and Sciences, Muş Alparslan University, 49100 Muş, Turkey

**Keywords:** 34D20, 34C11, stability, boundedness, Lyapunov functional, delay differential equations, fourth order, square integrability

## Abstract

In this paper, we give sufficient conditions for the boundedness, uniform asymptotic stability and square integrability of the solutions to a certain fourth order non-autonomous differential equations with delay by using Lyapunov’s second method. The results obtained essentially improve, include and complement the results in the literature.

## Introduction

In mathematical literature, ordinary differential equations have been studied for more than 300 years since the seventeenth century after the concepts of differentiation and integration were formulated by Newton and Leibniz. By means of ordinary differential equations, researchers can explain many natural phenomena like gravity, projectiles, wave, vibration, nuclear physics, and so on. In addition, in Newtonian mechanics, the system’s state variable changes over time, and the law that governs the change of the system’s state is normally described by an ordinary differential equation. The question concerning the stability of ordinary differential equations has been originally raised by the general problem of the stability of motion [1].

However, thereafter along with the development of technology, it have been seen that the ordinary differential equations cannot respond to the needs arising in sciences and engineering. For example, in many applications, it can be seen that physical or biological background of a modeling system shows that the change rate of the system’s current status often depends not only on the current state but also on the history of the system. This usually leads to the so-called retarded functional differential equations [2].

In particular, for more results on the stability, boundedness, convergence, etc. of ordinary or functional differential equations of fourth order, see the book of Reissig et al. [3] as a good survey for the works done by 1974 and the papers of Burton [4], Cartwright [5], Ezeilo [6–9], Harrow [10, 11], Tunç [12–18], Remili et al. [19–23], Wu [24] and others and the references therein. This information indicates the importance of investigating the qualitative properties of solutions of retarded functional differential equations of fourth order.

In this paper, we study the uniform asymptotic stability of the solutions for $p(t,x,x^{\prime},x^{\prime\prime}, x^{\prime\prime \prime })\equiv0$ and also square integrability and boundedness of solutions to the fourth order nonlinear differential equation with delay

1 $$ \begin{aligned}[b] &x^{(4)}+a(t) \bigl( g\bigl(x(t) \bigr)x^{\prime\prime}(t) \bigr) ^{\prime}+b(t) \bigl( q\bigl(x(t) \bigr)x^{\prime}(t) \bigr) ^{\prime}+c(t)f\bigl(x(t) \bigr)x^{\prime}(t) \\ &\quad{}+d(t)h\bigl(x(t-r)\bigr)=p\bigl(t,x,x^{\prime},x^{\prime \prime},x^{\prime\prime\prime } \bigr). \end{aligned} $$

For convenience, we get

$$ \theta_{1}(t)=g^{\prime}\bigl(x(t)\bigr)x^{\prime}(t), \qquad\theta_{2}(t)=q^{\prime}\bigl(x(t)\bigr)x^{\prime}(t), \qquad\theta_{3}(t)=f^{\prime }\bigl(x(t)\bigr)x^{\prime}(t). $$

We write (1) in the system form

2 $$ \begin{gathered} x^{\prime} =y, \\ y^{\prime} =z, \\ z^{\prime} =w, \\ w^{\prime} =-a(t)g(x)w- \bigl( b(t)q(x)+a(t)\theta_{1} \bigr) z- \bigl( b(t)\theta_{2}+c(t)f(x) \bigr) y-d(t)h(x) \\ \hphantom{w^{\prime} =}{}+d(t) \int_{t-r}^{t}h^{\prime}(x)y\,d\eta+p(t,x,y,z,w), \end{gathered} $$

where *r* is a positive constant to be determined later, the functions *a*, *b*, *c*, *d* are continuously differentiable functions and the functions *f*, *h*, *g*, *q*, *p* are continuous functions depending only on the arguments shown. Also derivatives $g^{\prime}(x)$, $q^{\prime}(x)$, $f^{\prime}(x)$ and $h^{\prime}(x)$ exist and are continuous. The continuity of the functions *a*, *b*, *c*, *d*, *p*, *g*, $g^{\prime}$, *q*, $q^{\prime}$, *f* and *h* guarantees the existence of the solutions of equation (1). If the right-hand side of system (2) satisfies a Lipschitz condition in $x(t)$, $y(t)$, $z(t)$, $w(t)$ and $x(t-r)$, and there exist solutions of system (2), then it is the unique solution of system (2).

Assume that there are positive constants $a_{0}$, $b_{0}$, $c_{0}$, $d_{0}$, $f_{0}$, $g_{0}$, $q_{0}$, $a_{1}$, $b_{1}$, $c_{1}$, $d_{1}$, $f_{1}$, $g_{1}$, $q_{1}$, *m*, *M*, *δ* and $\eta_{1}$ such that the following assumptions hold:

1. $0< a_{0}\leq a(t)\leq a_{1}$; $0< b_{0}\leq b(t)\leq b_{1}$; $0< c_{0}\leq c(t)\leq c_{1}$; $0< d_{0}\leq d(t)\leq d_{1}$ for $t\geq0$.
2. $0< f_{0}\leq f(x)\leq f_{1}$; $0< g_{0}\leq g(x)\leq g_{1}$; $0< q_{0}\leq q(x)\leq q_{1}$ for $x\in R$ and $0< m<\min \{ f_{0},g_{0},1 \} $, $M>\max \{ f_{1},g_{1},1 \} $.
3. $\frac{h(x)}{x}\geq\delta>0$ for $x\neq0$, $h(0)=0$.
4. $\int_{0}^{\infty} ( \vert a^{\prime}(t) \vert + \vert b^{\prime}(t) \vert + \vert c^{\prime}(t) \vert + \vert d^{\prime}(t) \vert ) \,dt<\eta_{1}$.
5. $\vert p(t,x,y,z,w) \vert \leq \vert e(t) \vert $.

Motivated by the results of references, we obtain some new results on the uniform asymptotic stability and boundedness of the solutions by means of Lyapunov’s functional approach. Our results differ from those obtained in the literature (see, [1–44] and the references therein). By this way, we mean that this paper has a contribution to the subject in the literature, and it may be useful for researchers working on the qualitative behaviors of solutions of functional differential equations of higher order. In view of all the mentioned information, the novelty and originality of the current paper can be checked.

## Preliminaries

We also consider the functional differential equation

3 $$ \overset{.}{x}=f(t,x_{t}),\quad x_{t}(\theta)=x(t+\theta ),-r\leq\theta\leq0,t\geq0, $$

where $f:I\times C_{H}\rightarrow \mathbb{R} ^{n}$ is a continuous mapping, $f(t,0)=0$, $C_{H}:=\{\phi\in(C[-r,0],\mathbb{R} ^{n}): \Vert \phi \Vert \leq H\}$, and for $H_{1}< H$, there exists $L(H_{1})>0$ with $\vert f(t,\phi) \vert < L(H_{1})$ when $\Vert \phi \Vert < H_{1}$.

### Lemma 1

[29]

*Let* $V(t,\phi):I\times C_{H}\rightarrow \mathbb{R} $ *be a continuous functional satisfying a local Lipschitz condition*, $V(t,0)=0 $, *and wedges* $W_{i}$ *such that*

(i) $W_{1}( \Vert \phi \Vert )\leq V(t,\phi)\leq W_{2}( \Vert \phi \Vert )$.
(ii) $V_{\text{(3)}}^{\prime}(t,\phi)\leq -W_{3}( \Vert \phi \Vert )$.

*Then the zero solution of equation* (3) *is uniformly asymptotically stable*.

## The main results

### Lemma 2

[35]

*Let* $h(0)=0$, $xh(x)>0$ ($x\neq0$) *and* $\delta (t)-h^{\prime}(x)\geq0$ ($\delta(t)>0$), *then* $2\delta(t)H(x)\geq h^{2}(x)$, *where* $H(x)=\int_{0}^{x}h(s)\,ds$.

### Theorem 1

*In addition to the basic assumptions imposed on the functions* *a*, *b*, *c*, *d*, *p*, *f*, *h*, *g* *and* *q*, *suppose that there are positive constants* $h_{0}$, $\delta_{0}$, $\delta_{1}$, $\eta_{2}$ *and* $\eta_{3}$ *such that the following conditions are satisfied*:

(i) $h_{0}-\frac{a_{0}m\delta_{0}}{d_{1}}\leq h^{\prime}(x)\leq \frac{h_{0}}{2}$ *for* $x\in R$.
(ii) $\delta_{1}=\frac{d_{1}h_{0}a_{1}M}{c_{0}m}+\frac {c_{1}M+\delta _{0}}{a_{0}m}< b_{0}q_{0}$.
(iii) $\int_{-\infty}^{+\infty} ( \vert g^{\prime }(s) \vert + \vert q^{\prime}(s) \vert + \vert f^{\prime }(s) \vert ) \,ds<\eta_{2}$.
(iv) $\int_{0}^{\infty} \vert e(t) \vert \,dt<\eta_{3}$.

*Then any solution* $x(t)$ *of equation* (1) *and its derivatives* $x^{\prime}(t)$, $x^{\prime\prime}(t)$ *and* $x^{\prime \prime\prime}(t)$ *are bounded and satisfy*

$$ \int_{0}^{\infty} \bigl( x^{\prime2}(s)+x^{\prime\prime 2}(s)+x^{\prime \prime\prime2}(s) \bigr) \,ds< \infty, $$

*provided that*

$$ r< \frac{2}{d_{1}h_{1}}\min\biggl\{ \frac{\varepsilon c_{0}m}{\alpha +2\beta +1}, \bigl[ b_{0}q_{0}- \delta_{1}-\varepsilon M(a_{1}+c_{1}) \bigr] , \frac{\varepsilon}{\alpha} \biggr\} . $$

### Proof

To prove the theorem, we define a Lyapunov functional

4 $$ W=W(t,x,y,z,w)=e^{\frac{-1}{\eta}\int_{0}^{t}\gamma(s)\,ds}V, $$

where

$$ \gamma(t)= \bigl\vert a^{\prime}(t) \bigr\vert + \bigl\vert b^{\prime }(t) \bigr\vert + \bigl\vert c^{\prime}(t) \bigr\vert + \bigl\vert d^{\prime }(t) \bigr\vert + \bigl\vert \theta _{1}(t) \bigr\vert + \bigl\vert \theta_{2}(t) \bigr\vert + \bigl\vert \theta_{3}(t) \bigr\vert , $$

and

$$ \begin{aligned} 2V &=2\beta d(t)H(x)+c(t)f(x)y^{2}+\alpha b(t)q(x)z^{2}+a(t)g(x)z^{2}+2 \beta a(t)g(x)yz \\ &\quad {}+ \bigl[ \beta b(t)q(x)-\alpha h_{0}d(t) \bigr] y^{2}- \beta z^{2}+\alpha w^{2}+2d(t)h(x)y+2\alpha d(t)h(x)z \\ &\quad {}+2\alpha c(t)f(x)yz+2\beta yw+2zw+\sigma \int_{-r}^{0} \int_{t+s}^{t}y^{2}(\gamma)\,d\gamma\,ds \end{aligned} $$

with $H(x)=\int_{0}^{x}h(s)\,ds$, $\alpha=\frac{1}{a_{0}m}+\varepsilon $, $\beta=\frac{d_{1}h_{0}}{c_{0}m}+\varepsilon$, and *η* are positive constants to be determined later in the proof. We can rearrange 2*V* as

$$\begin{aligned} 2V =&a(t)g(x) \biggl[ \frac{w}{a(t)g(x)}+z+\beta y \biggr] ^{2}+c(t)f(x) \biggl[ \frac{d(t)h(x)}{c(t)f(x)}+y+\alpha z \biggr] ^{2} \\ &{}+\frac{d^{2}(t)h^{2}(x)}{c(t)f(x)}+2d(t)H(x)+\sigma \int_{-r}^{0} \int_{t+s}^{t}y^{2}(\gamma)\,d\gamma \,ds+V_{1}+V_{2}+V_{3,} \end{aligned}$$

where

$$\begin{aligned}& V_{1} = 2d(t) \int_{0}^{x}h(s) \biggl[ \frac{d_{1}h_{0}}{c_{0}m}-2 \frac{d(t)}{c(t)f(x)}h^{\prime}(s) \biggr] \,ds, \\& V_{2} = \bigl[ \alpha b(t)q(x)-\beta-\alpha^{2}c(t)f(x) \bigr] z^{2}, \\& V_{3} = \bigl[ \beta b(t)q(x)-\alpha h_{0}d(t)-\beta ^{2}a(t)g(x) \bigr] y^{2}+ \biggl[ \alpha- \frac{1}{a(t)g(x)} \biggr] w^{2}. \end{aligned}$$

Let

5 $$ \varepsilon< \min\biggl\{ \frac{1}{a_{0}m},\frac{d_{1}h_{0}}{c_{0}m}, \frac{b_{0}q_{0}-\delta_{1}}{M(a_{1}+c_{1})} \biggr\} , $$

then

6 $$ \frac{1}{a_{0}m}< \alpha< \frac{2}{a_{0}m},\qquad\frac{d_{1}h_{0}}{c_{0}m}< \beta< 2 \frac{d_{1}h_{0}}{c_{0}m}. $$

By using conditions (A1)-(A3), (i)-(ii) and inequalities (5), (6), we have

$$\begin{aligned}& V_{1} \geq4d(t)\frac{d_{1}}{c_{0}m} \int_{0}^{x}h(s) \biggl[ \frac{h_{0}}{2}-h^{\prime}(s) \biggr] \,ds\geq0, \\& \begin{aligned} V_{2} &= \bigl( \alpha\bigl( b(t)q(x)-\beta a(t)- \alpha c(t)f(x) \bigr) +\beta\bigl(\alpha a(t)-1\bigr) \bigr) z^{2} \\ &\geq\alpha\biggl( b_{0}q_{0}-\frac{d_{1}h_{0}a_{1}}{c_{0}m}- \frac{c_{1}M}{a_{0}m}-(a_{1}+c_{1}M) \biggr) z^{2}+ \beta\biggl( \frac{1}{m}-1 \biggr) z^{2} \\ &\geq\alpha\bigl( b_{0}q_{0}-\delta_{1}-M ( a_{1}+c_{1} ) \bigr) z^{2}\geq0, \end{aligned} \end{aligned}$$

and

$$ \begin{aligned} V_{3} &\geq\beta\biggl( b_{0}q_{0}- \frac{\alpha}{\beta}h_{0}d_{1}-\beta a_{1}M \biggr) y^{2}+ \biggl( \alpha-\frac{1}{a_{0}m} \biggr) w^{2} \\ &\geq\beta\biggl( b_{0}q_{0}-\frac{c_{0}}{a_{0}}-a_{1} \frac{d_{1}h_{0}M}{c_{0}m}-(c_{0}m+a_{1}M) \biggr) y^{2}+w^{2} \\ &\geq\beta\bigl( b_{0}q_{0}-\delta_{1}-M(c_{1}+a_{1}) \bigr) y^{2}+w^{2}\geq0. \end{aligned} $$

Thus, it is clear from the above inequalities that there exists a positive constant $D_{0}$ such that

7 $$ 2V\geq D_{0}\bigl(y^{2}+z^{2}+w^{2}+H(x) \bigr). $$

From Lemma 2, (A3) and (i), it follows that there is a positive constant $D_{1}$ such that

8 $$ 2V\geq D_{1}\bigl(x^{2}+y^{2}+z^{2}+w^{2} \bigr). $$

In this way, *V* is positive definite. From (A1)-(A3), it is clear that there is a positive constant $U_{1}$ such that

9 $$ V\leq U_{1}\bigl(x^{2}+y^{2}+z^{2}+w^{2} \bigr). $$

From (iii), we have

10 $$ \begin{aligned}[b] &\int_{0}^{t} \bigl( \bigl\vert \theta _{1}(s) \bigr\vert + \bigl\vert \theta_{2}(s) \bigr\vert + \bigl\vert \theta_{3}(s) \bigr\vert \bigr) \,ds\\ &\quad = \int_{\alpha_{1}(t)}^{\alpha_{2}(t)} \bigl( \bigl\vert g^{\prime }(u) \bigr\vert + \bigl\vert q^{\prime}(u) \bigr\vert + \bigl\vert f^{\prime }(u) \bigr\vert \bigr) \,du \\ &\quad \leq \int_{-\infty}^{+\infty} \bigl( \bigl\vert g^{\prime}(u) \bigr\vert + \bigl\vert q^{\prime}(u) \bigr\vert + \bigl\vert f^{\prime}(u) \bigr\vert \bigr) \,du< \eta_{2}< \infty, \end{aligned} $$

where $\alpha_{1}(t)=\min \{ x(0),x(t) \} $ and $\alpha _{2}(t)=\max \{ x(0),x(t) \} $. From inequalities (5), (9) and (10), it follows that

11 $$ W\geq D_{2}\bigl(x^{2}+y^{2}+z^{2}+w^{2} \bigr), $$

where $D_{2}=\frac{D_{1}}{2}e^{-\frac{\eta_{1}+\eta_{2}}{\eta}}$. Also, it is easy to see that there is a positive constant $U_{2}$ such that

12 $$ W\leq U_{2}\bigl(x^{2}+y^{2}+z^{2}+w^{2} \bigr) $$

for all *x*, *y*, *z*, *w* and all $t\geq0$.

Now, we show that $\overset{.}{W}$ is a negative definite function. The derivative of the function *V* along any solution $( x(t),y(t),z(t),w(t) ) $ of system (2), with respect to *t*, is after simplifying

$$ 2\overset{.}{V}_{(2)}=-2\varepsilon c(t)f(x)y^{2}+V_{4}+V_{5}+V_{6}+V_{7}+V_{8}+V_{9}+2( \beta y+z+\alpha w)p(t,x,y,z,w), $$

where

$$\begin{aligned}& V_{4} =-2 \biggl( \frac{d_{1}h_{0}}{c_{0}m}c(t)f(x)-d(t)h^{\prime }(x) \biggr) y^{2}-2\alpha d(t) \bigl( h_{0}-h^{\prime}(x) \bigr) yz, \\& V_{5} =-2 \bigl( b(t)q(x)-\alpha c(t)f(x)-\beta a(t)g(x) \bigr) z^{2}, \\& V_{6} =-2 \bigl( \alpha a(t)g(x)-1 \bigr) w^{2}, \\& \begin{aligned} V_{7} &=2\alpha d(t)w \int_{t-r}^{t}h^{\prime}\bigl(x(\eta) \bigr)x^{\prime}(\eta)\,d\eta+2\beta d(t)y(t) \int_{t-r}^{t}h^{\prime}\bigl(x(\eta) \bigr)x^{\prime}(\eta)\,d\eta \\ &\quad{}+2d(t)z(t) \int_{t-r}^{t}h^{\prime}\bigl(x(\eta) \bigr)x^{\prime}(\eta)\,d\eta+\sigma ry^{2}(t)-\sigma \int_{t-r}^{t}y^{2}(\eta)\,d\eta, \end{aligned} \\& \begin{aligned} V_{8} &=-a(t)\theta_{1} \bigl( z^{2}+2\alpha zw \bigr) -b(t)\theta_{2} \bigl( \alpha z^{2}+2\alpha zw+\beta y^{2}+2yz \bigr) \\ &\quad{}+c(t)\theta_{3} \bigl( y^{2}+2\alpha yz \bigr) , \end{aligned} \\& \begin{aligned} V_{9} &=d^{\prime}(t) \bigl[ 2\beta H(x)-\alpha h_{0}y^{2}+2h(x)y+2\alpha h(x)z \bigr] \\ &\quad{}+c^{\prime}(t) \bigl[ f(x)y^{2}+2\alpha f(x)yz \bigr] +b^{\prime}(t) \bigl[ \alpha q(x)z^{2}+\beta q(x)y^{2} \bigr] \\ &\quad{}+a^{\prime}(t) \bigl[ g(x)z^{2}+2\beta g(x)yz \bigr] . \end{aligned} \end{aligned}$$

By regarding conditions (A1), (A2), (i), (ii) and inequality (6), (7), we have the following:

$$\begin{aligned} V_{4} \leq&-2 \bigl[ d(t)h_{0}-d(t)h^{\prime}(x) \bigr] y^{2}-2\alpha d(t)\bigl[ h_{0}-h^{\prime}(x) \bigr] yz \\ \leq&-2d(t) \bigl[ h_{0}-h^{\prime}(x) \bigr] y^{2}-2\alpha d(t) \bigl[ h_{0}-h^{\prime}(x) \bigr] yz \\ \leq&2d(t) \bigl[ h_{0}-h^{\prime}(x) \bigr] \biggl[ \biggl( y+\frac{\alpha }{2}z \biggr) ^{2}- \biggl( \frac{\alpha}{2}z \biggr) ^{2} \biggr] \\ \leq&\frac{\alpha^{2}}{2}d(t) \bigl[ h_{0}-h^{\prime}(x) \bigr] z^{2}. \end{aligned}$$

In that case,

$$\begin{aligned} V_{4}+V_{5} \leq&-2 \biggl[ b(t)q(x)-\alpha c(t)f(x)- \beta a(t)g(x)-\frac{\alpha^{2}}{4}d(t) \bigl[ h_{0}-h^{\prime}(x) \bigr] \biggr] z^{2} \\ \leq&-2 \biggl[ b_{0}q_{0}- \biggl( \frac{1}{a_{0}m}+ \varepsilon\biggr) c_{1}M- \biggl( \frac {d_{1}h_{0}}{c_{0}m}+\varepsilon \biggr) a_{1}M-\frac{\alpha^{2}}{4} ( a_{0}m\delta _{0} ) \biggr] z^{2} \\ \leq&-2 \biggl[ b_{0}q_{0}-\frac{M}{a_{0}m}c_{1}- \frac{d_{1}h_{0}a_{1}M}{c_{0}m}-\frac{\delta_{0}}{a_{0}m}-\varepsilon M ( a_{1}+c_{1} ) \biggr] z^{2} \\ \leq&-2 \bigl[ b_{0}q_{0}-\delta_{1}- \varepsilon M ( a_{1}+c_{1} ) \bigr] z^{2}\leq0, \end{aligned}$$

and

$$ V_{6}\leq-2 [ \alpha a_{0}m-1 ] w^{2}=-2 \varepsilon w^{2}\leq0. $$

By taking $h_{1}=\max \{ \vert h_{0}-\frac{a_{0}m\delta_{0}}{d_{1}} \vert ,\frac{h_{0}}{2} \} $, we get

$$ V_{7}\leq d_{1}h_{1}r\bigl(\alpha w^{2}+\beta y^{2}+z^{2}\bigr)+\sigma ry^{2}+ \bigl[ d_{1}h_{1}(\alpha+\beta+1)- \sigma\bigr] \int_{t-r}^{t}y^{2}(s)\,ds. $$

If we choose $\sigma=d_{1}h_{1}(\alpha+\beta+1)$, we have

$$ V_{7}\leq d_{1}h_{1}r \bigl[ \alpha w^{2}+(\alpha+2\beta+1)y^{2}+z^{2}\bigr] . $$

Thus, there exists a positive constant $D_{3}$ such that

$$ -2\varepsilon c(t)f(x)y^{2}+V_{4}+V_{5}+V_{6}+V_{7} \leq-2D_{3}\bigl(y^{2}+z^{2}+w^{2} \bigr). $$

From (8), and the Cauchy-Schwarz inequality, we obtain

$$\begin{aligned} V_{8} \leq&a(t) \vert \theta_{1} \vert \bigl( z^{2}+\alpha\bigl(z^{2}+w^{2}\bigr) \bigr) +b(t) \vert \theta_{2} \vert \bigl( \alpha z^{2}+ \alpha\bigl(z^{2}+w^{2}\bigr)+\beta y^{2}+y^{2}+z^{2} \bigr) \\ &{}+c(t) \vert \theta_{3} \vert \bigl( y^{2}+\alpha \bigl( y^{2}+z^{2} \bigr) \bigr) \\ \leq&\lambda_{1} \bigl( \vert \theta_{1} \vert + \vert \theta_{2} \vert + \vert \theta_{3} \vert \bigr) \bigl( y^{2}+z^{2}+w^{2}+H(x) \bigr) \\ \leq&2\frac{\lambda_{1}}{D_{0}} \bigl( \vert \theta_{1} \vert + \vert \theta_{2} \vert + \vert \theta_{3} \vert \bigr) V, \end{aligned}$$

where $\lambda_{1}=\max \{ a_{1} ( 1+\alpha ) ,b_{1} ( 1+2\alpha+\beta ) ,c_{1} ( 1+\alpha ) \} $. Using condition (iii) and Lemma 2, we can write

$$ h^{2}(x)\leq h_{0}H(x), $$

hereby,

$$\begin{aligned} \vert V_{9} \vert \leq& \bigl\vert d^{\prime}(t) \bigr\vert \bigl[ 2\beta H(x)+\alpha h_{0}y^{2}+h^{2}(x)+y^{2}+ \alpha\bigl( h^{2}(x)+z^{2} \bigr) \bigr] \\ &{}+ \bigl\vert c^{\prime}(t) \bigr\vert \bigl[ y^{2}+ \alpha\bigl( y^{2}+z^{2} \bigr) \bigr] + \bigl\vert b^{\prime}(t) \bigr\vert \bigl[ \alpha z^{2}+\beta y^{2} \bigr] \\ &{}+ \bigl\vert a^{\prime}(t) \bigr\vert \bigl[ z^{2}+2 \beta\bigl( y^{2}+z^{2} \bigr) \bigr] \\ \leq&\lambda_{2} \bigl[ \bigl\vert a^{\prime}(t) \bigr\vert + \bigl\vert b^{\prime}(t) \bigr\vert + \bigl\vert c^{\prime}(t) \bigr\vert + \bigl\vert d^{\prime}(t) \bigr\vert \bigr] \bigl( y^{2}+z^{2}+w^{2}+H(x) \bigr) \\ \leq&2\frac{\lambda_{2}}{D_{0}} \bigl[ \bigl\vert a^{\prime }(t) \bigr\vert + \bigl\vert b^{\prime}(t) \bigr\vert + \bigl\vert c^{\prime }(t) \bigr\vert + \bigl\vert d^{\prime}(t) \bigr\vert \bigr] V, \end{aligned}$$

such that $\lambda_{2}=\max \{ 2\beta+(\alpha+1)h_{0},\alpha h_{0}+1,\alpha+1 \} $. By taking $\frac{1}{\eta}=\frac{1}{D_{0}}\max \{ \lambda_{1},\lambda_{2} \} $, we obtain

13 $$\begin{aligned} \overset{.}{V}_{(2)} \leq&-D_{3} \bigl( y^{2}+z^{2}+w^{2} \bigr) + ( \beta y+z+\alpha w ) p(t,x,y,z,w) \\ &{}+\frac{1}{\eta} \bigl( \bigl\vert a^{\prime}(t) \bigr\vert + \bigl\vert b^{\prime}(t) \bigr\vert + \bigl\vert c^{\prime}(t) \bigr\vert + \bigl\vert d^{\prime}(t) \bigr\vert + \vert \theta _{1} \vert + \vert \theta_{2} \vert + \vert \theta _{3} \vert \bigr) V. \end{aligned}$$

From (A4), (A5),(iii), (10), (11), (13) and the Cauchy-Schwarz inequality, we get

14 $$\begin{aligned} \overset{.}{W}_{(2)} =& \biggl( \overset{.}{V}_{(2)}- \frac{1}{\eta }\gamma(t)V \biggr) e^{-\frac{1}{\eta}\int_{0}^{t}\gamma(s)\,ds} \\ \leq& \bigl( -D_{3} \bigl( y^{2}+z^{2}+w^{2} \bigr) + ( \beta y+z+\alpha w ) p(t,x,y,z,w) \bigr) e^{-\frac{1}{\eta }\int_{0}^{t}\gamma(s)\,ds} \end{aligned}$$

15 $$\begin{aligned} \leq& \bigl( \beta \vert y \vert + \vert z \vert +\alpha \vert w \vert \bigr) \bigl\vert p(t,x,y,z,w) \bigr\vert \\ \leq&D_{4} \bigl( \vert y \vert + \vert z \vert + \vert w \vert \bigr) \bigl\vert e(t) \bigr\vert \\ \leq&D_{4} \bigl( 3+y^{2}+z^{2}+w^{2} \bigr) \bigl\vert e(t) \bigr\vert \\ \leq&D_{4} \biggl( 3+\frac{1}{D_{2}}W \biggr) \bigl\vert e(t) \bigr\vert \\ \leq&3D_{4} \bigl\vert e(t) \bigr\vert +\frac{D_{4}}{D_{2}}W \bigl\vert e(t) \bigr\vert , \end{aligned}$$

where $D_{4}=\max \{ \alpha,\beta,1 \} $. Integrating (15) from 0 to *t* and using condition (iv) and the Gronwall inequality, we have

16 $$\begin{aligned} W \leq&W \bigl( 0,x(0),y(0),z(0),w(0) \bigr) +3D_{4}\eta _{3} \\ &{}+\frac{D_{4}}{D_{2}} \int_{0}^{t}W \bigl( s,x(s),y(s),z(s),w(s) \bigr) \bigl\vert e(s) \bigr\vert \,ds \\ \leq& \bigl( W \bigl( 0,x(0),y(0),z(0),w(0) \bigr) +3D_{4}\eta _{3} \bigr) e^{\frac{D_{4}}{D_{2}}\int_{0}^{t} \vert e(s) \vert \, ds} \\ \leq& \bigl( W \bigl( 0,x(0),y(0),z(0),w(0) \bigr) +3D_{4}\eta _{3} \bigr) e^{\frac{D_{4}}{D_{2}}\eta_{3}}=K_{1}< \infty. \end{aligned}$$

Because of inequalities (11) and (16), we write

17 $$ \bigl( x^{2}+y^{2}+z^{2}+w^{2} \bigr) \leq\frac{1}{D_{2}}W\leq K_{2}, $$

where $K_{2}=\frac{K_{1}}{D_{2}}$. Clearly, (17) implies that

$$ \bigl\vert x(t) \bigr\vert \leq\sqrt{K_{2}},\qquad\bigl\vert y(t) \bigr\vert \leq\sqrt{K_{2}},\qquad\bigl\vert z(t) \bigr\vert \leq\sqrt{K_{2}},\qquad\bigl\vert w(t) \bigr\vert \leq \sqrt{K_{2}}\quad\text{for all }t\geq0. $$

Hence

18 $$ \begin{gathered} \bigl\vert x(t) \bigr\vert \leq\sqrt{K_{2}},\qquad \bigl\vert x^{\prime }(t) \bigr\vert \leq\sqrt{K_{2}},\qquad \bigl\vert x^{\prime\prime }(t) \bigr\vert \leq\sqrt{K_{2}},\\ \bigl\vert x^{\prime\prime\prime }(t) \bigr\vert \leq\sqrt{K_{2}}\quad\text{for all }t\geq0. \end{gathered} $$

Now, we prove the square integrability of solutions and their derivatives. We define $F_{t}=F(t,x(t),y(t),z(t),w(t))$ as

$$ F_{t}=W+\rho \int_{0}^{t} \bigl( y^{2}(s)+z^{2}(s)+w^{2}(s) \bigr) \,ds, $$

where $\rho>0$. It is easy to see that $F_{t}$ is positive definite since $W=W(t,x,y,z,w)$ is already positive definite. Using the estimate

$$ e^{-\frac{\eta_{1}+\eta_{2}}{\eta}}\leq e^{-\frac{1}{\eta}\int_{0}^{t}\gamma(s)\,ds}\leq1 $$

by (15), we have the following:

19 $$ \begin{aligned}[b] \overset{.}{F_{t}}_{(2)} &\leq-D_{3} \bigl( y^{2}(t)+z^{2}(t)+w^{2}(t) \bigr) e^{-\frac{\eta_{1}+\eta_{2}}{\eta}} \\ &\quad {}+D_{4} \bigl( \bigl\vert y(t) \bigr\vert + \bigl\vert z(t) \bigr\vert + \bigl\vert w(t) \bigr\vert \bigr) \bigl\vert e(t) \bigr\vert \\ &\quad {}+\rho\bigl( y^{2}(t)+z^{2}(t)+w^{2}(t) \bigr). \end{aligned} $$

By choosing $\rho=D_{3}e^{-\frac{\eta_{1}+\eta_{2}}{\eta}}$, we obtain

20 $$ \begin{aligned}[b] \overset{.}{F_{t}}_{(2)} &\leq D_{4} \bigl( 3+y^{2}(t)+z^{2}(t)+w^{2}(t) \bigr) \bigl\vert e(t) \bigr\vert \\ &\leq D_{4} \biggl( 3+\frac{1}{D_{2}}W \biggr) \bigl\vert e(t) \bigr\vert \\ &\leq 3D_{4} \bigl\vert e(t) \bigr\vert +\frac{D_{4}}{D_{2}}F_{t} \bigl\vert e(t) \bigr\vert . \end{aligned} $$

Integrating inequality (20) from 0 to *t* and using again the Gronwall inequality and condition (iv), we get

21 $$\begin{aligned} F_{t} \leq&F_{0}+3D_{4}\eta_{3}+\frac{D_{4}}{D_{2}} \int_{0}^{t}F_{s}\vert e(s) \vert \,ds \\ \leq& ( F_{0}+3D_{4}\eta_{3} ) e^{\frac{D_{4}}{D_{2}}\int_{0}^{t} \vert e(s) \vert \,ds} \\ \leq& ( F_{0}+3D_{4}\eta_{3} ) e^{\frac{D_{4}}{D_{2}}\eta _{3}}=K_{3}< \infty. \end{aligned}$$

Therefore,

$$ \int_{0}^{\infty}y^{2}(s)\,ds< K_{3}, \qquad \int_{0}^{\infty }z^{2}(s)\,ds< K_{3}, \qquad \int_{0}^{\infty}w^{2}(s)\,ds< K_{3}, $$

which implies that

22 $$ \int_{0}^{\infty} \bigl[ x^{\prime}(s) \bigr] ^{2}\,ds< K_{3},\qquad \int_{0}^{\infty} \bigl[ x^{\prime\prime}(s) \bigr] ^{2}\,ds< K_{3},\qquad \int_{0}^{\infty} \bigl[ x^{\prime\prime\prime}(s) \bigr] ^{2}\,ds< K_{3}, $$

which completes the proof of the theorem. □

### Remark 1

If $p(t,x,y,z,w)\equiv0$, similarly to the above proof, inequality (14) becomes

$$\begin{aligned} \overset{.}{W_{(2)}} =& \biggl( \overset{.}{V}_{(2)}- \frac{1}{\eta}\gamma(t)V \biggr) e^{-\frac{1}{\eta}\int_{0}^{t}\gamma(s)\,ds} \\ \leq&-D_{3}\bigl(y^{2}+z^{2}+w^{2} \bigr)e^{-\frac{1}{\eta}\int_{0}^{t}\gamma(s)\,ds} \\ \leq&-\mu\bigl(y^{2}+z^{2}+w^{2}\bigr), \end{aligned}$$

where $\mu=D_{3}e^{-\frac{\eta_{1}+\eta_{2}}{\eta}}$. It can also be observed that the only solution of system (2) for which $\overset{.}{W_{(2)}}(t,x,y,z,w)=0$ is the solution $x=y=z=w=0$. The above discussion guarantees that the trivial solution of equation (1) is uniformly asymptotically stable, and the same conclusion as in the proof of the theorem can be drawn for square integrability of solutions of equation (1).

### Example 1

We consider the following fourth order nonlinear differential equation with delay:

23 $$ \begin{aligned}[b] &x^{(4)}+\bigl(e^{-2t}\sin 3t+2\bigr) \biggl( \biggl(\frac{5x+2e^{x}+2e^{-x}}{e^{x}+e^{-x}}\biggr)x^{\prime\prime} \biggr) ^{\prime} \\ &\qquad{}+\biggl(\frac{\sin2t+11t^{2}+11}{t^{2}+1}\biggr) \biggl( \biggl(\frac{\sin x+9e^{x}+9e^{-x}}{e^{x}+e^{-x}} \biggr)x^{\prime} \biggr) ^{\prime} \\ &\qquad{}+\bigl(e^{-t}\sin t+3\bigr) \biggl(\frac{x\cos x+x^{4}+1}{x^{4}+1} \biggr)x^{\prime}+\biggl(\frac{\sin ^{2}t+t^{2}+1}{5t^{2}+5}\biggr) \biggl( \frac{x(t-\frac {1}{17})}{x^{2}(t-\frac{1}{17})+1} \biggr) \\ &\quad=\frac{2\sin t}{t^{2}+1+(x^{\prime}x^{\prime\prime })^{2}+(xx^{\prime \prime\prime})^{2}} \end{aligned} $$

by taking $g(x)=\frac{5x+2e^{x}+2e^{-x}}{e^{x}+e^{-x}}$, $q(x)=\frac{\sin x+9e^{x}+9e^{-x}}{e^{x}+e^{-x}}$, $f(x)=\frac{x\cos x+x^{4}+1}{x^{4}+1}$, $h(x)=\frac {x}{x^{2}+1}$, $a(t)= e^{-2t}\sin3t+2$, $b(t)=\frac{\sin2t+11t^{2}+11}{t^{2}+1}$, $c(t)=e^{-t}\sin t+3$, $d(t)=\frac{\sin^{2}t+t^{2}+1}{5t^{2}+5}$, $r=\frac {1}{17}$ and $p(t,x,x^{\prime}x^{\prime\prime}, x^{\prime\prime\prime })=\frac{2\sin t}{t^{2}+1+(x^{\prime}x^{\prime\prime})^{2}+(xx^{\prime \prime\prime})^{2}}$.

We obtain easily the following: $g_{0}=0.33$, $g_{1}=3.7$, $f_{0}=0.5$, $f_{1}=1.5$, $q_{0}=8.5$, $q_{1}=9.5$, $a_{0}=1$, $a_{1}=3$, $b_{0}=10$, $b_{1}=12$, $c_{0}=2$, $c_{1}=4$, $d_{0}=0.2$, $d_{1}=0.3$, $m=0.3$, $M=3.8$, $h_{0}=2$, $\alpha=\frac{23}{6}$, $\beta=\frac{3}{2}$, $\delta _{0}=\frac{17}{8}$ and $\delta_{1}=69.15$. Also we have

$$\begin{aligned}& \begin{aligned} \int_{-\infty}^{\infty} \bigl\vert g^{\prime}(x) \bigr\vert \,dx &=5 \int_{-\infty}^{\infty} \biggl\vert \frac{1}{e^{x}+e^{-x}}+x \frac{e^{-x}-e^{x}}{ ( e^{x}+e^{-x} ) ^{2}} \biggr\vert \,dx \\ &\leq5 \int_{-\infty}^{0} \biggl\vert \frac{1}{e^{x}+e^{-x}}-x \frac{e^{-x}-e^{x}}{ ( e^{x}+e^{-x} ) ^{2}} \biggr\vert \,dx \\ & {}+5 \int_{0}^{\infty} \biggl\vert \frac{1}{e^{x}+e^{-x}}-x \frac{e^{-x}-e^{x}}{ ( e^{x}+e^{-x} ) ^{2}} \biggr\vert \,dx \\ &=5\pi, \end{aligned} \\& \begin{aligned} \int_{-\infty}^{\infty} \bigl\vert q^{\prime}(x) \bigr\vert \,dx &= \int_{-\infty}^{\infty} \biggl\vert \frac{ ( e^{x}+e^{-x} ) \cos x- ( e^{x}-e^{-x} ) \sin x}{ ( e^{x}+e^{-x} ) ^{2}} \biggr\vert \,dx \\ &\leq \int_{-\infty}^{\infty} \biggl\vert \frac{1}{e^{x}+e^{-x}}+x\frac{e^{x}-e^{-x}}{ ( e^{x}+e^{-x} ) ^{2}} \biggr\vert \,dx \\ &=\pi, \end{aligned} \\& \begin{aligned} \int_{-\infty}^{\infty} \bigl\vert f^{\prime}(x) \bigr\vert \,dx &= \int_{-\infty}^{\infty} \biggl\vert \frac{\cos x}{x^{4}+1}-4x^{4}\frac{\cos x}{ ( x^{4}+1 ) ^{2}}+-x\frac{\sin x}{x^{4}+1}\biggr\vert \,dx \\ &\leq \int_{-\infty}^{\infty} \biggl\vert \frac{5}{x^{4}+1}+ \frac{x^{2}}{x^{4}+1} \biggr\vert \,dx \\ &=6\sqrt{2}\pi, \end{aligned} \\& \begin{aligned} \int_{0}^{\infty} \bigl\vert p\bigl(t,x,x^{\prime},x^{\prime\prime },x^{\prime \prime\prime} \bigr) \bigr\vert \,dt &= \int_{0}^{\infty} \biggl\vert \frac{2\sin t}{t^{2}+1+(x^{\prime}x^{\prime\prime})^{2}+(xx^{\prime\prime\prime })^{2}} \biggr\vert \,dt \\ &\leq \int_{0}^{\infty} \biggl\vert \frac{2\sin t}{t^{2}+1} \biggr\vert \,dt \\ &\leq \int_{0}^{\infty}\frac{2}{t^{2}+1}\,dt \\ &=\pi, \end{aligned} \\& \begin{aligned} \int_{0}^{\infty} \bigl\vert a^{\prime}(t) \bigr\vert \,dt &= \int_{0}^{\infty} \bigl\vert -2e^{-2t}\sin 3t+3e^{-2t}\cos3t \bigr\vert \,dt \\ &\leq \int_{0}^{\infty}5e^{-2t}\,dt \\ &=\frac{5}{2}, \end{aligned} \\& \begin{aligned} \int_{0}^{\infty} \bigl\vert b^{\prime}(t) \bigr\vert \,dt &= \int_{0}^{\infty} \biggl\vert \frac{2\cos2t}{t^{2}+1}-2t \frac{\sin2t}{( t^{2}+1 ) ^{2}} \biggr\vert \,dt \\ &\leq \int_{0}^{\infty}\frac{3}{t^{2}+1}\,dt \\ &=\frac{3\pi}{2}, \end{aligned} \\& \begin{aligned} \int_{0}^{\infty} \bigl\vert c^{\prime}(t) \bigr\vert \,dt &= \int_{0}^{\infty} \bigl\vert -e^{-t}\sin t+e^{-t}\cos t \bigr\vert \,dt \\ &\leq \int_{0}^{\infty}2e^{-t}\,dt \\ &=2, \end{aligned} \\& \begin{aligned} \int_{0}^{\infty} \bigl\vert d^{\prime}(t) \bigr\vert \,dt &= \int_{0}^{\infty} \biggl\vert \frac{2\sin t\cos t}{5t^{2}+5}-2t \frac{\sin ^{2}t}{ ( 5t^{2}+5 ) ^{2}} \biggr\vert \,dt \\ &\leq\frac{11}{25} \int_{0}^{\infty}\frac{1}{t^{2}+1}\,dt \\ &=\frac{11\pi}{50}. \end{aligned} \end{aligned}$$

Consequently,

$$ \begin{gathered} \int_{-\infty}^{+\infty} \bigl( \bigl\vert g^{\prime}(s) \bigr\vert + \bigl\vert q^{\prime}(s) \bigr\vert + \bigl\vert f^{\prime}(s) \bigr\vert \bigr) \,ds< \infty, \\ \int_{0}^{\infty} \bigl( \bigl\vert a^{\prime}(t) \bigr\vert + \bigl\vert b^{\prime}(t) \bigr\vert + \bigl\vert c^{\prime}(t) \bigr\vert + \bigl\vert d^{\prime}(t) \bigr\vert \bigr) \,dt< \infty. \end{gathered} $$

Thus all the assumptions of Theorem 1 hold. This shows that every solution of equation (23) is bounded and square integrable.

## Conclusion

A class of nonlinear retarded functional differential equations of fourth order is considered. Sufficient conditions are established guaranteeing the uniform asymptotic stability of the solutions for $p(t,x,x^{\prime },x^{\prime\prime},x^{\prime\prime\prime})\equiv0$ and also square integrability and boundedness of solutions of equation (1) with delay. In the proofs of the main results, we benefit from Lyapunov’s functional approach. The results obtained essentially improve, include and complement the results in the literature.
